# Effect of heme oxygenase-1 on the expression of interferon-stimulated genes

**DOI:** 10.1186/s12950-025-00467-5

**Published:** 2025-10-09

**Authors:** Patryk Chudy, Katarzyna Bednarczyk, Eryk Chatian, Wojciech Krzeptowski, Agata Szade, Krzysztof Szade, Monika Żukowska, Jan Wolnik, Grzegorz Sokołowski, Anna Grochot-Przęczek, Alicja Józkowicz, Witold N. Nowak

**Affiliations:** 1 Department of Medical Biotechnology, Faculty of Biochemistry, Biophysics and Biotechnology, Jagiellonian University, Gronostajowa 7, Kraków, 30-387 Poland; 2 Doctoral School of Exact and Natural Sciences, Jagiellonian University, Kraków, Poland; 3 Laboratory of Stem Cell Biology, Faculty of Biochemistry, Biophysics and Biotechnology, Jagiellonian University, Kraków, Poland; 4 August Chełkowski Institute of Physics, Faculty of Science and Technology, University of Silesia in Katowice, Chorzów, Poland; 5 Silesia LabMed: Centre for Research and Implementation, Medical University of Silesia in Katowice, Katowice, Poland; 6 Department of Drug Addiction Pharmacology, Laboratory of Neuropharmacology and Epigenetics, Maj Institute of Pharmacology, Polish Academy of Sciences, Kraków, Poland

## Abstract

Heme oxygenase-1 (HO1, *Hmox1*) degrades excess heme and is considered an anti-oxidative and anti-inflammatory enzyme. Our previous studies in *Hmox1* knockout mice revealed the induction of interferon-stimulated genes (ISGs) in all cell types analyzed, despite unchanged interferon production. Here, we sought to determine whether this induction is driven by intrinsic cellular mechanisms or extrinsic cues at the organismal level, and to identify the pathway underlying HO1-dependent ISG regulation. To this end, we analyzed how ISG expression changes in cultured cells exposed to stressors typical of *Hmox1* knockout mice. Using murine wild-type and *Hmox1*-deficient (Hmox1 KO) fibroblasts, we found that under control conditions, the expression of most tested ISGs was independent of cellular HO1 status. We next examined the effects of extrinsic stressors, including hemolytic, oxidative, genotoxic, and replication stress, proinflammatory TNFα, and endogenous heme overload. TNFα, which is upregulated in *Hmox1* knockout mice, was the sole and universal inducer of ISGs in both wild-type and Hmox1 KO fibroblasts. Unexpectedly, the response of Hmox1 KO cells to exogenous TNFα was weakened, likely due to impaired NF-κB activity and reduced nuclear retention of the p65 subunit. A similar decrease we observed for STAT1. Additionally, the presence of the TREX1 exonuclease in the nucleus pointed to compromised nuclear envelope integrity in HO-deficient cells. Notably, HO1 colocalizes with PARP1, a protein involved in envelope maintenance and regulation of cytoplasmic-nuclear transport. Inhibition of PARP1 with olaparib dampened TNFα-induced nuclear accumulation of p65 and STAT1 in wild-type cells, but not in Hmox1 KO counterparts. In summary, the inflammation observed in *Hmox1*-deficient mice appears to be the main cell-extrinsic driver of ISG induction in vivo. Despite this, the inflammatory response to exogenous TNFα is intrinsically attenuated in Hmox1 KO cells, likely due to decreased nuclear retention of NF-κB and STAT1.

**Supplementary Information:**

The online version contains supplementary material available at 10.1186/s12950-025-00467-5.

## Introduction

Heme oxygenase-1 (HO1, encoded by the *Hmox1* gene) is an enzyme that degrades heme and affects various cellular processes, including DNA replication, cell cycle progression, and cell differentiation [1–3]. In contrast to the constitutive HO2 (encoded by *Hmox2*), HO1 is an inducible isoform, that is upregulated under stressful conditions. HO1 is best known for its cytoprotective, antioxidant, anti-inflammatory, and proangiogenic functions. These effects are mediated either by heme degradation products (biliverdin, Fe^2+^ ions, and carbon monoxide) [4], or by regulation of heme availability [3, 5], or by direct binding of HO1 protein to transcription factors [6]. However, modulation of HO1 expression or activity can have widely varying effects depending on the cellular context [7].

**Fig. 1:**
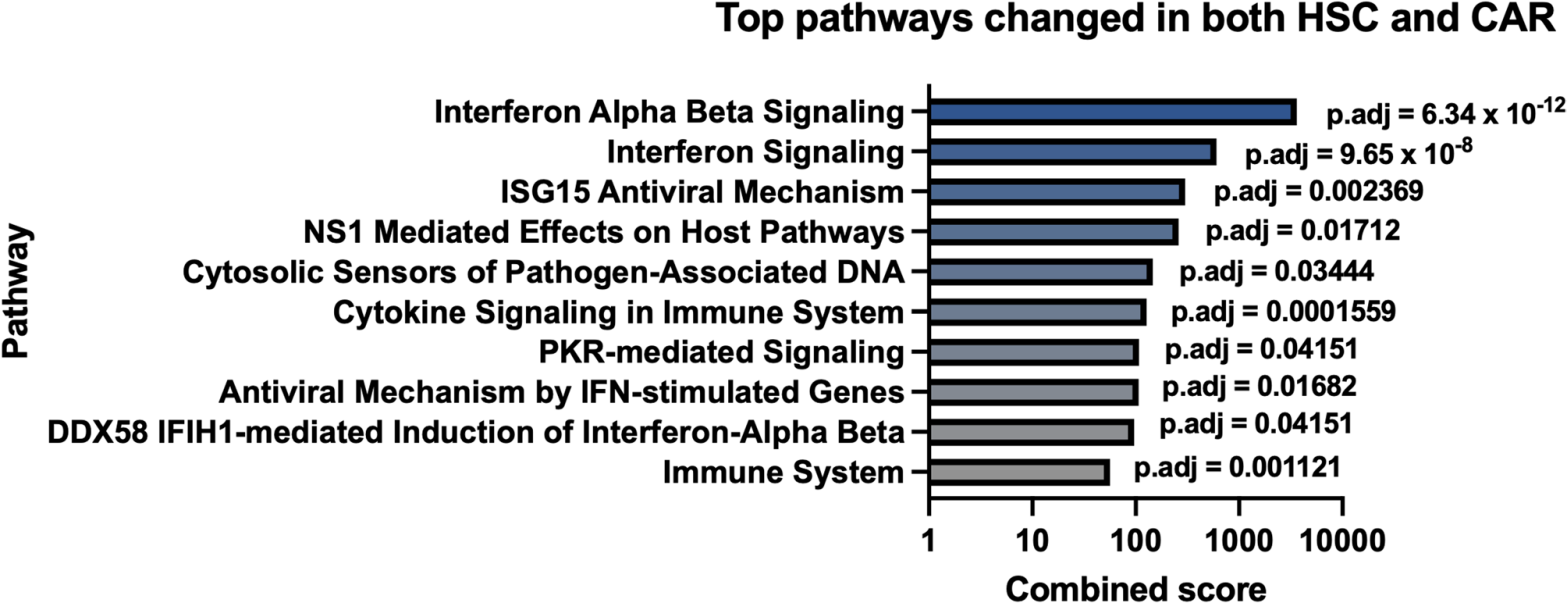
Pathways enriched in mouse HSC and CAR using Enrichr tool on 27 genes affected in both HSC and CAR with p.adj.<0.1 Data from [8]

Our team has shown that HO1 in the bone marrow niche is necessary for protecting hematopoietic stem cells (HSCs) from premature aging [8]. By comparing the transcriptomes of wild-type and Hmox1 KO primary mouse HSCs, bone marrow-derived endothelial cells (ECs), and CXCL12-abundant reticular cells (CARs) [8], as well as muscle satellite cells (SCs) [9], we found that there is no single gene expression pattern consistently associated with *Hmox1* deficiency. However, gene List enrichment analysis Based on the 27 genes altered in both HSCs and CARs Hmox1 KO showed that interferon signaling is potentially affected in Hmox1-deficient cells, with Interferon Alpha/Beta Signaling being the most significantly changed Reactome pathway (padj < 6.3 × 10^−12^) (Fig. 1). Among top four upregulated genes in *Hmox1* KO HSCs were *Ifi44*,* Ifi204*, and *Ifi27*, while in Hmox1 KO CARs the top four hits included *Oas1g*,* Oas1a*, and *Ifi27l2a* [8]. More broadly, in HSC, CAR, EC, and SC cells isolated from Hmox1 KO mice, we observed increased expression of interferon-stimulated genes (ISGs). These included interferon-regulatory factors (IRFs), interferon-induced proteins (IFIs), interferon-induced proteins with tetratricopeptide repeats (IFITs), and 2’−5’-oligoadenylate synthetases (OASs), whereas the expression of genes encoding interferons and their receptors remained unaffected [8, 9].

Type I interferons are a family of cytokines that form part of the antiviral defense system. This group includes IFN-β and a set of closely related IFN-α proteins [10, 11]. All IFN-I signals are mediated through a common receptor, the type I interferon receptor (IFNAR), which is a heterodimer composed of IFNAR1 and IFNAR2 subunits. IFN-α is produced at particularly high levels by mononuclear phagocytes, whereas IFN-β is primarily expressed by non-hematopoietic cells [12]. The main stimulus for IFN-I expression is a viral infection. The initial recognition of viral nucleic acids and the first wave of IFN-I production are predominantly mediated by pattern recognition receptors (PRRs), such as endosomal Toll-like receptor (TLR) 7 and 9 [13]. Subsequently, viral replication products are detected by cytosolic sensors, including DDX58 or DDX60 helicase [14].

Upon activation of TLR7 and TLR9, signal is transduced via IRFs, primarily IRF3 and IRF7, which translocate from the cytoplasm to the nucleus to promote transcription of IFN-α and IFN-β [15, 16]. TLR7 and TLR9 receptors can also activate the NF-κB transcription factor, initiating an inflammatory response [17]. Type I interferons bind to IFNAR, thereby activating the receptor-associated tyrosine kinases TYK2 and JAK1. This leads to the phosphorylation of STAT1 and STAT2 proteins. STATs bind to IRF9 and form the heterotrimeric transcription factor ISGF3, which recognizes IFN-stimulated response elements (ISRE) in target genes and promotes their transcription [18]. A group of these genes, together with IRF7 and IRF9 – master regulators of IFN-I dependent cellular responses – are strongly upregulated in different *Hmox1*-deficient primary cell types [8, 9]. This upregulation might be expected to occur in a cell-autonomous manner, as HO1 protein has been shown to directly interact with IRF3 and modulate its activity [19, 20]. However, this mechanism has not yet been directly confirmed.

Additionally, our recent work on the protective role of HO1 in replication stress showed that HO1 modulates the expression and function of PARP1 [3] – an enzyme that catalyzes the transfer of ADP-ribose moieties from nicotinamide adenine dinucleotide (NAD^+^) to a plethora of target proteins in response to DNA damage, cellular stress, and inflammation, in a process known as PARylation [21]. PARP1, along with other members of the PARP family, modulates the IFN-I response [22]. PARylation also enhances NF-κB activity and promotes its nuclear retention [22, 23]. On the other hand, mono-ADP-ribosylation (MARylation) by PARP10, another member of the family, negatively regulates NF-κB signaling [24]. Finally, PARylation is essential for the activity of STAT1, which forms both ISFG3 complexes [25] and homodimers that activate IRF1 and IRF8 [26]. Therefore, we suppose that the cellular effects of HO1 deficiency on IFN-I response genes may involve the HO1-PARP1 axis.

It should be emphasized, however, that comparisons of gene expression profiles in primary cells isolated directly from wild-type and *Hmox1* knockout (KO) mice [5, 8] do not allow for distinguishing between cell-autonomous effects and responses to external signals originating from the *Hmox1*-deficient microenvironment or the organism as a whole. In both mice and humans, the lack of HO1 is associated with increased susceptibility to oxidative stress, an enhanced DNA damage response, and the development of progressive inflammatory disease – all of which may influence ISGs [8, 19, 27, 28]. Therefore, distinguishing intrinsic from extrinsic effects on the gene expression profile would require analyzing ISG levels in cells cultured in vitro under homeostatic conditions, followed by testing their response to defined extrinsic stressors.

We aimed to elucidate whether dysregulation of interferon-stimulated genes also occurs in *Hmox1*-deficient cells cultured in vitro and how ISG expression changes in response to stressors typical of *Hmox1* deficiency.

## Materials and methods

### Animals

C57BL/6J×FVB Hmox1^KO^ (Hmox1 KO) and C57BL/6J×FVB Hmox1^WT^ (WT) mice were bred in the animal facility of the Faculty (Breeder Registry No. 078). The animals were used exclusively for tissue collection; therefore, no additional Ethics Committee approval was required. Mice were housed in individually ventilated cages under specific pathogen-free conditions, with ad libitum access to food and water.

### Primary fibroblast isolation and cell culture

WT and Hmox1 KO mice bred in our animal facility were used for fibroblast isolation. Mice were euthanized via CO_2_ inhalation. Fibroblasts were isolated from mouse tails by digestion in collagenase II (2.5 mg/mL in DPBS, Gibco) for 90 min, followed by filtration through a 70 μm cell strainer (Biologix). Cell cultures were maintained under standard conditions at 37 °C in a humidified atmosphere with 5% CO_2_. Fibroblasts were cultured in DMEM High-Glucose medium (Biowest) supplemented with 10% fetal bovine serum (FBS, EurX), antibiotics (100 IU/mL penicillin and 100 µg/mL streptomycin, Sigma-Aldrich) and fibroblast growth factor 2 (FGF2; 10 ng/mL, PeproTech). Cells were cultured for a maximum of three passages in T-25 flasks (Falcon) and then seeded into 6-well or 24-well plates (Falcon) on glass coverslips.

In experiments, cells were treated with: 10 ng/mL tumor necrosis factor-α (TNFα, a kind gift from Dr. Krystyna Stalińska, Department of Cell Biotechnology, FBBB, Jagiellonian University, Kraków, Poland); 10 ng/mL aphidicolin (APH, A0781-1MG, Sigma-Aldrich); 0.25 µM etoposide (ETO, E1383-250MG, Sigma-Aldrich); 350 µM 5-aminolevulinic acid (ALA, A7793-500MG, Sigma-Aldrich); 20 mg/mL hemoglobin (Hb, H2500, Sigma-Aldrich); 100 µM H_2_O_2_ (95321-500ML, Sigma-Aldrich); 10 µM NF-κB pathway inhibitor (INH14, MedChem Express); 10 µM AP-1 pathway inhibitor (T5224, MedChem Express); or 0.1 µM olaparib (HY-10162, MedChem Express).

### Induced pluripotent stem cells (iPSCs)

Mouse Hmox1 KO and WT iPSCs were obtained earlier by other members of our team by reprogramming Hmox1 KO or WT primary skin fibroblasts [29, 30].

Double knockout (dKO) *Hmox1*^−/−^ *Hmox2*^−/−^ iPSCs were generated from Hmox1-KO iPSCs using pSpCas9(BB)−2 A-Puro plasmid [31] with an sgRNA encoding insert targeting *Hmox2* (top: CAC CGG CCT TCC GGT GTA GCT CCG T, bottom: AAA CAC GGA GCT ACA CCG GAA GGC C). Then, we prepared lentiviral vectors carrying *Hmox1* expression cassette with added nuclear localization signal (NLS) or nuclear export signal (NES). Lentiviral vectors harboring *Hmox1-NLS* or *Hmox1-NES* genes were cloned using a pLVX-EF1α-IRES-puro backbone plasmid (Takara). Vectors were produced in HEK293 cells transfected with pMD2.G plasmid (VSV-G envelope expressing plasmid; addgene, #12259), psPAX2 plasmid (2nd generation lentiviral packaging plasmid; addgene, #12260) and pLVX-EF1α-Hmox1-NLS-IRES-puro or pLVX-EF1α-Hmox1-NES-IRES-puro plasmids using PEI Max (Polysciences). Medium with vectors was collected from packaging cells 48 h and 78 h after transfection, centrifuged at 1000 *g* for 10 min, filtered through 0.45 μm syringe filter (VWR), and used directly on iPS dKO cells mixed with iPS medium 1:1 and with 0.5 µg/mL polybrene (Sigma-Aldrich). Medium was changed after 24 h. Transduced cells were selected using 0.5 µg/mL puromycin (Sigma-Aldrich).

All iPSCs were cultured in DMEM High-Glucose medium containing 20% FBS, 1% Non-Essential Amino Acids (Life Technologies), 0.1 mM β-mercaptoethanol (Life Technologies), 1000 U/mL mouse leukemia inhibitory factor (mLIF, Millipore), and antibiotics, in 6-well plates coated with Geltrex (LDEV-Free Reduced Growth Factor Basement Membrane Matrix (Gibco) dissolved 1:100 in DMEM/F12 (Biowest).

### Immunofluorescence staining

Cells grown on glass coverslips covered with 1% Geltrex were fixed in 4% Pierce methanol-free formaldehyde (Thermo Scientific) for 10 min at room temperature, followed by two washes with PBS (Lonza). Fixed cells were permeabilized in 0.2% Triton X-100 in PBS (PBS-Tx) for 10 min and blocked in 10% normal donkey serum (Sigma-Aldrich) in 0.1% PBS-Tx for 30 min at room temperature. Next, cells were incubated overnight at 4 °C with primary antibodies diluted 1:200 in 0.1% PBS-Tx supplemented with 1% donkey serum. After three PBS washes, secondary antibodies diluted 1:400 in 0.1% PBS-Tx with 1% donkey serum were applied for 1 h at room temperature. Following additional PBS washes, nuclei were counterstained with 0.5 µg/mL DAPI (Sigma-Aldrich) for 10 min at room temperature. Coverslips were mounted in Fluorescence Mounting Medium (Dako) and allowed to dry before imaging. Negative controls omitting the primary antibody were included for each protein. Cells were analyzed using the Axio Observer Z1 microscope (Zeiss) with 63x/1.4 Oil DIC m27 objective or using Leica DMI6000 with 40x/0.75 objective.

Fluorescence microscopy image analysis was conducted using ImageJ software. For proteins distributed throughout the entire cell, the mean fluorescence intensity was quantified for each image, as precise delineation of individual cell boundaries proved challenging. In the case of nuclear-localized staining, a binary mask was generated from the DAPI channel using the “Make Binary” and “Analyze Particles…” functions. This mask was subsequently applied to the corresponding channel of the target protein to extract nuclear fluorescence measurements.

### Antibodies

The following antibodies were used: anti-HO-1 (ADI-SPA-894, Enzo); anti-TNFR1 (BS-2941R, Thermo Scientific); anti-phospho-IκB-α (Ser32/36) (9246, Cell Signaling Technology); anti-NF-κB p65 (51–0500, Thermo Scientific); anti-phospho-NF-κB p65 (Ser536) (MA5-15160, Thermo Scientific); anti-STAT1 (PA5-95442, Thermo Scientific); anti-STAT2 (MA5-42463, Thermo Scientific); anti-lamin A/C (4C11, Abcam); anti-exportin-1/CRM1 (D6V7N) (46249T, Cell Signaling Technology); anti-tubulin hFAB rhodamine (12004166, Bio-Rad); anti-rabbit StarBright Blue 700 (12004161, Bio-Rad); anti-rabbit HRP-conjugated antibody (7074P2, Cell Signaling Technology); and anti-rabbit Alexa Fluor 488 (A21206, Thermo Scientific).

### Analysis of NR4A1 in mouse hematopoietic stem cells (HSCs)

Bone marrow was isolated from femurs and tibias of 6-month-old male mice as described previously [8]. The bone marrow was filtered through a 100 μm cell strainer and centrifuged at 670 x *g* for 10 min at 4 °C. The cell pellet was resuspended in RBC lysis buffer (0.15 mol/L NH_4_Cl, 10 mmol/L KHCO_3_, 0.1 mmol/L EDTA) and incubated for 7 min at room temperature. After dilution with PBS containing 2% FBS, cells were centrifuged again and the resulting pellet was resuspended in 100 µL of PBS with 2% FBS. Mouse HSCs (Lin^−^c-Kit^+^Sca-1^+^ [LKS] CD150^+^ CD48^−^), multipotent progenitors (MPPs; LKS CD150^−^ CD48^−^), and granulocyte-monocyte progenitors (GMPs; LKS CD150^−^CD48^+^) were stained using the following antibody clones: anti-mouse CD3, clone 17A2; anti-mouse Ly-6G/Ly-6 C, clone RB6-8C5; anti-mouse CD11b, clone M1/70; anti-mouse CD45R/B220, clone RA3-6B2; anti-mouse TER-119/erythroid cells, clone Ter-119; anti-mouse-CD150, clone TC15-12F12.2; anti-mouse-CD48, clone HM-48-1 (all BioLegend); and anti-mouse-Ly6A/E (Sca-1), clone D7; and anti-mouse-CD117 (c-Kit), clone 2B8 (both from eBioscience). Following surface staining, cells were fixed and permeabilized using BD IntraSure kit (BD Biosciences) and stained intracellularly with anti-Nurr77 (NR4A1) antibody (clone JM59-11, Thermo Scientific), followed by donkey anti-rabbit AlexaFluor^®^568 secondary antibody (Thermo Scientific). The NR4A1 signal was collected in the PE-Texas Red channel on a BD LSR Fortessa flow cytometer. A secondary-only control (omitting the primary Nurr77/NR4A1 antibody) was used to establish gating.

### Detection of oxidative stress

General oxidative stress was assessed using the CellROX™ Deep Red Reagent (Thermo Fisher Scientific). Cells were cultured in 12-well plates. As a positive control, cells were treated with 200 µM tert-butyl hydroperoxide (TBHP) for 30 min. For reactive oxygen species (ROS) detection, CellROX™ Deep Red reagent was added to each sample at a final concentration of 500 nM and incubated for 30 min at 37 °C. Fluorescence was analyzed using LSR Fortessa flow cytometer (Becton Dickinson).

### Lipid peroxidation

Click-iT Lipid Peroxidation (LAA) Kit for Imaging – Alexa Fluor 488 (C10446, Thermo Fisher Scientific) was used, following the manufacturer’s instructions. Cells were cultured in 24-well plates on round glass coverslips and stimulated with 50 µM linoleamide alkyne (LAA) for 24 h. Cumene hydroperoxide (CH) was used as a positive control. After stimulation, cells were fixed with 4% Pierce methanol-free formaldehyde (Thermo Scientific) for 10 min at room temperature and washed twice with PBS. Permeabilization was performed with 0.1% PBS-Tx for 10 min at room temperature. Lipid peroxidation was detected by incubating the coverslips with 50 µL of Click-iT reaction cocktail for 30 min at room temperature. Samples were then washed three times with PBS and counterstained with DAPI (0.5 µg/mL; Sigma-Aldrich). Fluorescence was detected using a Leica DM6B fluorescence microscope with a PL Fluotar L 20x/0.40 objective.

### Reverse transcription and real-time PCR

Total RNA was isolated using the RNeasy Mini Kit (Qiagen) and reverse transcribed with the QuantiTect Reverse Transcription Kit (Qiagen), which includes genomic DNA removal. Gene expression was analyzed by real-time PCR on a StepOnePlus thermocycler (Applied Biosystems) using SYBR Green JumpStart Taq ReadyMix (Sigma-Aldrich) and specific primers. Primer sequences were as follows: *Eef2* (F: GCG GTC AGC ACA ATG GCA TA, R: GAC ATC ACC AAG GGT GTG CAG); *Irf1* (F: GGA TAT GGA AAG GGA CAT AAC, R: ATA AGG TCT TCG GCT ATC TTC); *Irf7* (F: TAA GGT GTA CGA ACT TAG CC, R: TAC TGC AGA ACC TGT GTG); *Irf9* (F: CTA CTT CTG TAG AGA TTT GGC, R: GAT GAG ATT CTC TTG GCT ATG); *Ifitm3* (F: AAC TTC TGA GAA ACC GAA AC, R: ATC TCA GCC ACC TCA TAT TC); *Adar1* (F: CAT CAG GTT TCT CTA CAG TG, R: CTG CAG GAT TTG TCA AAG AG); *Oas1g* (F: TCA ATG TCG TGT GTG ATT TC, R: CTG GTG AGA TTG TTA AGG AAC); *Oasl1* (F: CTC CTC TGT ATC TAC TGG AC, R: CCA CTA TGT CCC ATC TGT AG). *Eef2* served as a reference gene.

### Immunoblotting

Cultured cells were detached with TrypLE (Gibco), washed in cold PBS (Lonza) and centrifuged at 400 x *g* for 10 min. Cell pellets were lysed in Pierce RIPA buffer (Thermo Fisher Scientific) supplemented with protease inhibitors (Complete Protease Inhibitor Cocktail, Merck) and incubated for 5 min at 4 °C with agitation. Lysates were clarified by centrifugation at 8000 x *g* for 10 min at 4 °C. Protein concentration was determined using a BCA assay kit (Thermo Fisher Scientific) to ensure equal loading (10 µg of proteins). Samples were separated on 4–20% Mini-PROTEAN TGX Precast Protein Gels (BioRad) and transferred to nitrocellulose membranes using the Trans-Blot Turbo RTA Mini Transfer Kit (0.2 μm pore, Bio-Rad) in buffer containing 20% ethanol. Membranes were blocked with EveryBlot Blocking Buffer (BioRad) for 5 min and incubated overnight at 4 °C with primary antibodies. After three washes with TBST, membranes were incubated for 1 h at room temperature with appropriate secondary antibodies: anti-rabbit-HRP, anti-rabbit StarBright Blue 700, or anti-tubulin hFAB rhodamine (BioRad), all diluted in EveryBlot blocking buffer. After five additional TBST washes, HRP substrate (Bio-Rad) was applied when HRP-conjugated antibodies were used. Signals were detected by chemiluminescence or fluorescence using a ChemiDoc MP imaging system (Bio-Rad).

### Trans-AM ELISA

Cells were seeded in 6-well plates at a density of 100,000 cells/well and stimulated with 10 ng/mL TNFα for 30 min. Cells were then detached using TrypLE (Gibco), washed with cold PBS (Lonza), and centrifuged at 400 x *g* for 10 min. Nuclear fractions were isolated from cell pellets using the Cell Fractionation Kit – Standard (ab109719, Abcam). Transcriptional activity of NF-κB was assessed using the TransAM NF-κB p65 assay (40096, Active Motif). Briefly, 2 µg of nuclear extract was added per well of a 96-well plate pre-coated with oligonucleotides containing the NF-κB consensus binding site in Complete Binding Buffer, and incubated for 1 h at room temperature. After three washes, the wells were incubated for 1 h with an anti–NF-κB p65 antibody (1:1000 in Antibody Binding Buffer), followed by three washes and a 1-hour incubation with an HRP-conjugated secondary antibody (1:1000 in Antibody Binding Buffer). After four final washes, the colorimetric reaction was developed using Developing Solution for 5 min, followed by Stop Solution. Absorbance was measured at 450 nm with a reference wavelength of 655 nm using an Infinite M200 microplate reader (TECAN).

### NF-κB reporter assay

Cells were seeded in a 24-well plates at a density of 50 000 cells/well and transfected with 200 ng/well of pNL3.2.NF-κB-RE [NlucP/NF-κB-RE/Hygro] Vector (N1111, Promega) using Lipofectamine 2000 (11668027, Thermo Scientific) according to the manufacturer’s protocol. After 24 h of incubation at 37 °C, cells were treated with 100 ng/mL TNFα for 5 h. Cells were then detached using TrypLE (Gibco), centrifuged at 400 x *g* for 10 min, and resuspended in culture medium. For detection of luciferase activity, an equal volume of Nano-Glo Luciferase Assay reagent (N1110, Promega) was added directly to the cell suspension in black 96-well plates (Perkin Elmer). After 3 min of incubation at room temperature, luminescence was measured using an Infinite M200 microplate reader (TECAN).

### Proximity ligation assay (PLA)

To visualize the in-situ interaction between p65 and CRM1 proteins, we performed a proximity ligation assay using the Duolink In Situ Detection Reagents Orange kit (Sigma-Aldrich), following the manufacturer’s protocol. Cells were cultured on glass coverslips pre-covered with 1% Geltrex LDEV-Free Reduced Growth Factor Basement Membrane Matrix (Gibco), fixed with 4% Pierce methanol-free formaldehyde (Thermo Scientific) for 10 min at room temperature, and permeabilized with 0.2% PBS-Tx for additional 10 min. After blocking in Duolink Blocking Solution (Sigma-Aldrich) for 1 h at 37 °C, cells were incubated with primary antibodies: anti-p65 (sc-8008, Santa Cruz) and anti-CRM1 (46249T, Cell Signaling Technology), each diluted 1:200 in Duolink Antibody Diluent (Sigma-Aldrich). Following washing in buffer A (10 mM Tris, 150 mM NaCl, 0.05% Tween 20), cells were incubated with PLA probes: anti-mouse PLUS and anti-rabbit MINUS (Sigma-Aldrich). Next, cells were again washed twice in buffer A, and ligation was performed using Duolink ligase (diluted 1:40 in ligation buffer) for 30 min at 37 °C, followed by amplification with Duolink polymerase (diluted 1:80 in an amplification buffer) for 100 min at 37 °C. Cells were then washed in buffer B (200 mM/L Tris, 100 mM/L NaCl), counterstained with DAPI (0.5 µg/mL, Sigma-Aldrich), mounted in Fluorescence Mounting Medium (Dako), and allowed to dry before imaging. Negative controls were prepared by using primary and secondary antibodies only. Fluorescence images were acquired using a Zeiss Axio Observer Z1 microscope equipped with a 63×/1.4 oil immersion DIC objective.

### RNA Seq data

We analyzed our previously published data available at in the BioProject database, accession no. PRJNA562450 [8].

### Cytokine quantification

Serum levels of INFγ, interleukin-1β (IL-1β), interleukin-10 (IL-10), monocyte chemoattractant protein-1 (MCP1) and TNFα protein concertation in WT and Hmox1 KO mice were measured using Luminex assay (MILLIPLEX MAP Mouse Cytokine/Chemokine Premixed 32 Plex, Mouse Cytokine/Chemokine Magnetic Bead Premixed 32 Plex and the custom assay panel, Millipore), following the manufacturer’s instructions. Serum samples were diluted 1:1 in Assay Buffer and incubated overnight at 4 °C with premixed beads. Signal acquisition was performed using FLEXMAP 3D system (Millipore).

### ELISA

Serum concentrations of INFα/β in WT and Hmox1 KO mice were measured using an ELISA kit (R&D Systems), according to the manufacturer’s instructions. Absorbance was measured on a TECAN infinite M200 microplate reader (TECAN).

### Statistical analysis

All experiments were performed in duplicate or triplicate and were repeated independently at least three times unless stated otherwise. Data were analyzed with GraphPad Prism 8.0. The statistical tests used are indicated in the figure legends. Bar graphs show mean ± SEM. The violin plots display the median along with the first and third quartiles. Significance is defined as follows: ns – nonsignificant, *p < 0.05; **p < 0.01; ***p < 0.001.

## Results

### HO1 deficiency leads to upregulation of Ifi27 both in vivo and in vitro

**Fig. 2:**
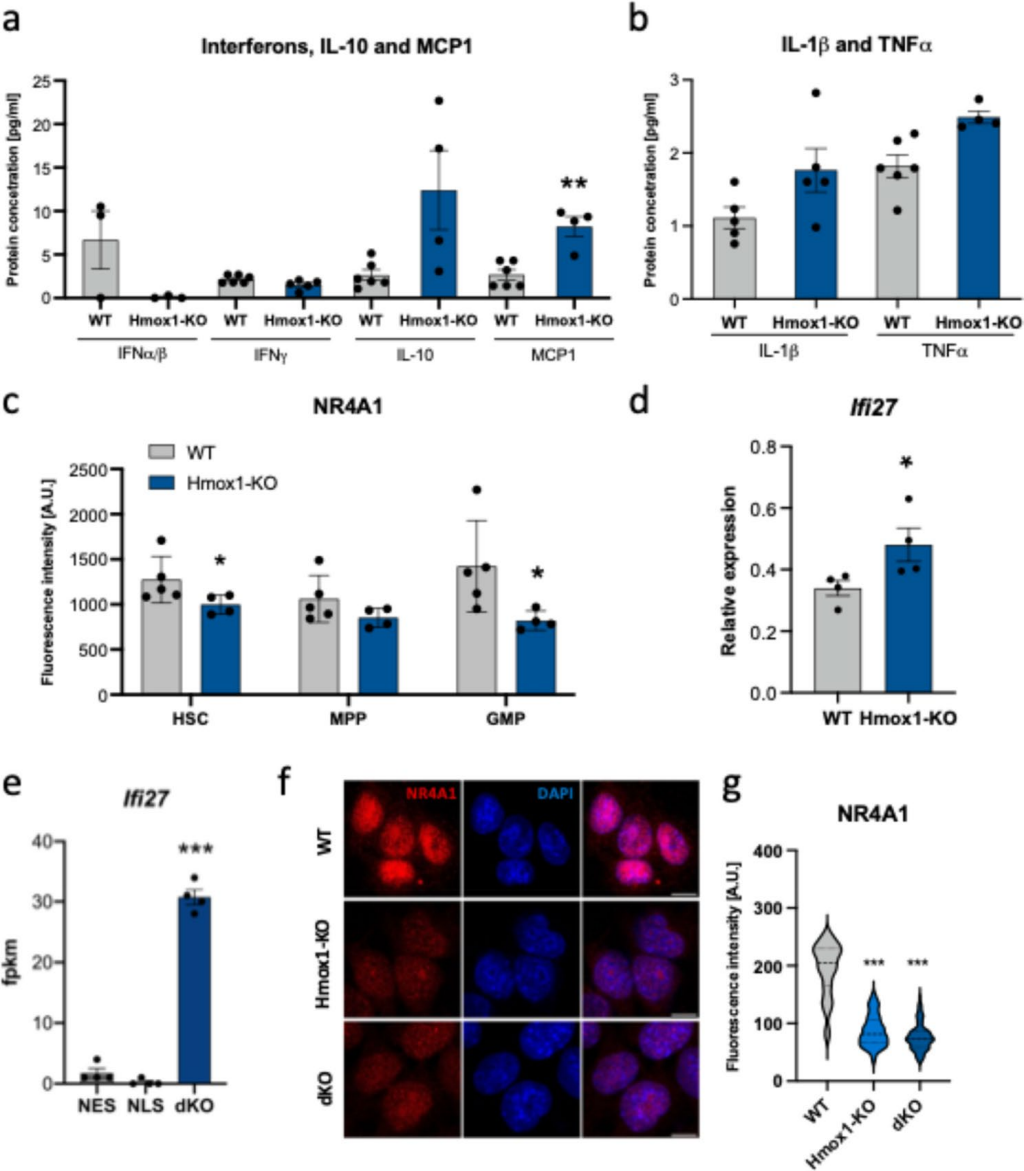
Concentrations of IFNα, IFNɣ, IL-10, and MCP1 (**a**), and IL-1β and TNFα (**b**) in the blood serum of WT and Hmox1 KO mice (ELISA and Luminex assay; *N* = 3–6; unpaired t-test). **c** NR4A1 protein levels in HSCs, MPPs and GMPs isolated from WT and Hmox1 KO mice (flow cytometry analysis; *N* = 5; unpaired t-test). **d** *Ifi27* gene expression in WT and Hmox1 KO fibroblasts under control conditions (qPCR; *N* = 4; unpaired t-test; * - Hmox1 KO vs. WT). **e** *Ifi27* gene expression in dKO, NES, and NLS iPSCs cultured under control conditions (RNA-seq; *N* = 4; ANOVA with Holm-Sidak’s test; * - dKO vs. NES and NLS). **f** Representative images of NR4A1 immunofluorescence staining in WT, Hmox1 KO, and Hmox1 KO/2 iPSCs. Scale Bar: 10 μm, **g** statistical analysis of total NR4A1 protein level. Kruskal-Wallis

In our earlier studies we observed increased mRNA expression of numerous interferon-stimulated genes (ISGs), but not interferons and their receptors [8, 9]. These analyses were performed in cell types that are not considered primary interferon producers, namely HSCs, ECs, and CARs from the bone marrow, and satellite cells from the muscle. We also measured interferon concentration in the blood serum. Consistently, serum levels of INFα/β and INFγ remained very low, near the detection limits, and did not differ significantly between genotypes (Fig. 2A). This confirms that the production of interferons in *Hmox1*-deficient mice was not enhanced, despite the upregulated expression of ISGs. In the same animals, we detected a trend toward increased concentration of interleukin-10 (IL-10) and a significant elevation of monocyte chemoattractant protein-1 (MCP1) – cytokines typically induced by IFNα and IFNγ, respectively [32]. These findings may further support ISG activation. Additionally, we observed a tendency for increased concentrations of interleukin-1β (IL-1β) and a significant elevation of tumor necrosis factor-α (TNFα), consistent with the proinflammatory phenotype of Hmox1 KO mice (Fig. 2B).

Importantly, changes in ISG expression appear to have functional significance. The gene whose expression was universally upregulated across nearly all *Hmox1*-deficient cells was *Ifi27*, along with its paralog *Ifi27l2a*. The IFI27 protein localizes to the inner membrane of the nuclear envelope and is involved, among other functions, in the export of the nuclear receptor NR4A1 (also known as NUR77) from the nucleus to the cytoplasm in an exportin-1 (CRM1)-dependent manner. Cytoplasmic export leads to proteasomal degradation of NR4A1 and inhibition of NR4A1-dependent signaling pathways. This mechanism is particularly important in HSCs, where NR4A1 is a key regulator of quiescence and metabolism [33]. It can therefore be assumed that increased *Ifi27* expression would be associated with reduced NR4A1 protein levels. Indeed, we observed a decrease in NR4A1 protein levels in HSCs and granulocyte-monocyte progenitors (GMPs), with a similar trend in multipotent progenitors (MPPs) isolated from *Hmox1*-deficient mice (Fig. 2C).

To test whether *Ifi27* is also upregulated in other cell types, we used fibroblasts isolated from Hmox1 KO mice and cultured in vitro for several passages. RT-PCR analysis revealed a slight but statistically significant increase in *Ifi27* expression in fibroblasts lacking HO1 (Fig. 2D). In addition, we used induced pluripotent stem cells (iPSCs) in which the endogenous *Hmox1* and *Hmox2* genes had been deleted and subsequently engineered to express the cytoplasmic or nuclear form of HO1 [3]. Here, we also observed strong upregulation of *Ifi27* in the absence of HO1 (Fig. 2E). Similarly to the results obtained in hematopoietic cells (Fig. 2C), the absence of HO1 – or both HO1 and HO2 – was associated with reduced NR4A1 protein levels without affecting its cellular localization, confirming the functional significance of changes in IFI27 expression (Fig. 2F, G). The increased expression of *Ifi27* not only in *Hmox1*-deficient cells directly isolated from mice, but also in those cultured in vitro, may suggest the existence of intrinsic regulatory mechanisms.

### In cells grown under homeostatic conditions, the expression of many ISGs is independent of HO1 status

**Fig. 3:**
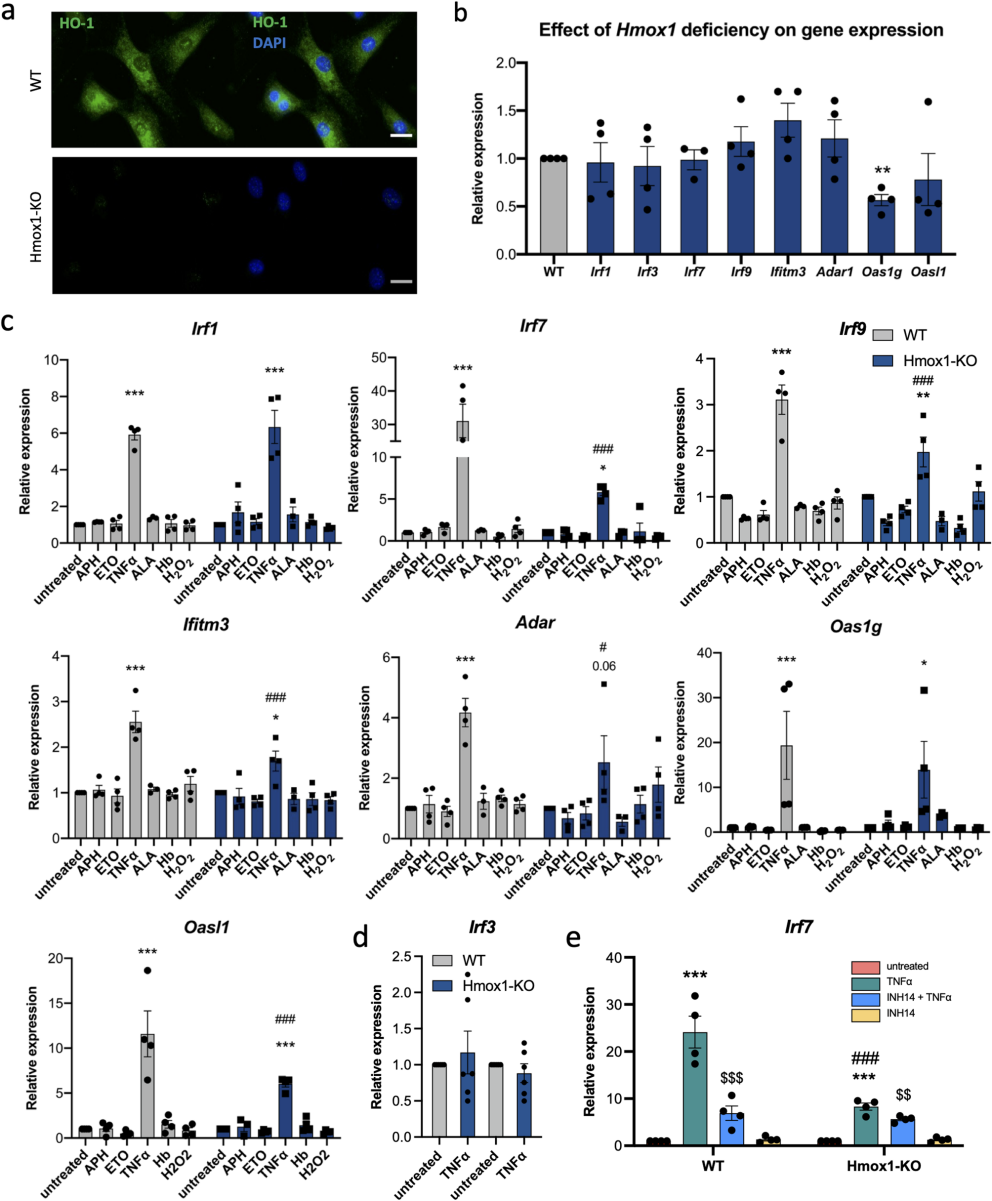
Expression of ISGs in WT and Hmox1 KO fibroblasts cultured in vitro. **a** Representative images of HO1 immunofluorescence staining (green) and nuclei counterstained with DAPI (blue) in WT and Hmox1 KO fibroblasts. Scale Bar: 20 μm. **b** Relative expression of ISGs in Hmox1 KO fibroblasts (blue) compared to WT fibroblasts (gray) under control conditions (qRT-PCR; *N* = 4; ANOVA with Holm-Sidak’s test). **c** Relative expression of *Irf1*,* Irf7*,* Irf9*,* Ifitm3*,* Adar1*,* Oas1g*,* Oasl1* in WT and Hmox1 KO fibroblasts, untreated or exposed to stressors: aphidicolin (APH, 0.01 µg/mL); etoposide (ETO, 0.25 µM); TNFα (10 ng/mL); 5-aminolevulinic acid (ALA, 350 µM), hemoglobin (Hb, 20 mg/mL), and H_2_O_2_ (100 µM) for 24 h. (qRT-PCR; *N* = 3–4; two-way ANOVA. * - treated vs. untreated, # - Hmox1 KO vs. WT. Gene expression in treated cells was normalized to control conditions for each genotype separately. **d** Relative expression of *Irf3* in WT and Hmox1 KO fibroblasts treated with TNFα (10 ng/mL) for 24 h (qRT-PCR; *N* = 6; two-way ANOVA). **e** Relative expression of *Irf7* in WT and Hmox1 KO fibroblasts untreated or treated with TNFα (10 ng/ml) and INH14 (inhibitor of NF-κB pathway, 10 µM), both individually or in combination for 24 h (qRT-PCR; *N* = 3; two-way ANOVA. * - TNFα vs. untreated, $ - INH14 + TNFα vs. untreated, # - Hmox1 KO vs. WT)

All subsequent experiments were conducted using fibroblasts cultured in vitro, which exhibit relatively high levels of HO1 protein under control conditions (Fig. 3A). In contrast to wild-type (WT) cells, in fibroblasts lacking *Hmox1* (Hmox1 KO), HO1 protein was undetectable (Fig. 3A). Notably, Hmox1 KO cells did not differ from their wild type counterparts in terms of total reactive oxygen species (ROS) levels (Fig. S1A) or lipid peroxidation (Fig. S1B). This means that the lack of HO1 does not induce oxidative stress in our experimental setting.

First, we examined the impact of genotype on the basal expression of several ISGs, namely *Irf1*, *Irf7*, *Irf9*, *Ifitm3*, *Adar*, *Oas1g* and *Oasl1*. All these genes were upregulated in HSCs and some other cell types isolated from Hmox1 KO mice [8, 9]. However, in fibroblasts cultured in vitro under control conditions, unlike *Ifi27* (Fig. 2D), none of the tested genes showed increased expression in *Hmox1*-deficient cells (Fig. 3B). This indicates that the upregulation of ISGs in vivo is likely driven by some external stressor(s).

### Expression of ISGs is upregulated by TNFα

In the next step, we stimulated fibroblasts with compounds known to induce various types of stress previously described in *Hmox1*-deficient cells: aphidicolin (APH – replication stress), etoposide (ETO – genotoxic stress), TNFα (inflammatory response), 5-aminolevulinic acid (ALA – enhanced heme synthesis and overload), hemoglobin (Hb – hemolytic stress), and H_2_O_2_ (oxidative stress) for 24 h.

Among all tested compounds, only TNFα significantly increased the expression of *Irf1*,* Irf7*,* Irf9*,* Ifitm3*,* Adar*,* Oas1g* and *OasL1*. Moreover, TNFα acted as a universal stressor, inducing the expression of all tested ISGs in both WT and Hmox1 KO fibroblasts in vitro (Fig. 3C). These findings suggest that the upregulation of ISGs observed in various primary cells in vivo is predominantly driven by extrinsic factors and may be a consequence of the pre-inflammatory state typical of Hmox1 KO mice, particularly the increased production of TNFα. It is worth noting that the expression of *Irf3*, which is not regulated at the transcriptional level [34] and which was not induced in vivo (data not shown), did not change in vitro in response to TNFα (Fig. 3D).

### Response to TNFα is reduced in Hmox1 KO cells

The regulation of two genes, *Irf1* and *Oas1g*, by TNFα was comparable regardless of HO1 status. Surprisingly, however, the induction of *Irf7*,* Irf9*,* Ifitm3*,* Adar*, and *OasL1* was significantly lower in the absence of HO1 (Fig. 3C). This suggests that, unlike under baseline conditions (which are proinflammatory in Hmox1 KO mice), the inflammatory response to exogenous TNFα is more pronounced in the presence of HO1.

The induction of ISGs may be regulated by NF-κB [35, 36], which directly controls. the expression of *Irf7* [37]. On the other hand, it has been suggested that NF-κB activity may be modulated by HO1, both via its metabolic products and through a direct protein-protein interaction between HO1 and the p65 subunit of NF-κB [38]. Therefore, to investigate the mechanism underlying the attenuated response to TNFα, we first examined whether NF-κB ​​was indeed involved in the induction of ISGs in our cellular model.

To this end, we treated WT and Hmox1 KO fibroblasts with TNFα in the presence or absence of INH14, a specific inhibitor of the NF-κB pathway, and analyzed the expression of *Irf7* (Fig. 3E). INH14 inhibited the response to TNFα stimulation, confirming the involvement of the NF-κB-dependent mechanism. However, the inhibitory effect was much stronger in WT cells, where activation was reduced by 71% compared to only 32% in Hmox1 KO cells. Thus, the induction of *Irf7* expression in response to TNFα was markedly stronger in WT cells, but in the presence of the NF-κB inhibitor, the response was similar in both genotypes (*p* < 0.001 and *p* > 0.99, respectively; two-way ANOVA). These findings may suggest that the enhanced response to TNFα in the presence of HO1 may results from more efficient NF-κB signaling, and that this pathway may be impaired in *Hmox1*-deficient cells.

### Lack of HO1 affects the nuclear localization of p65

**Fig. 4:**
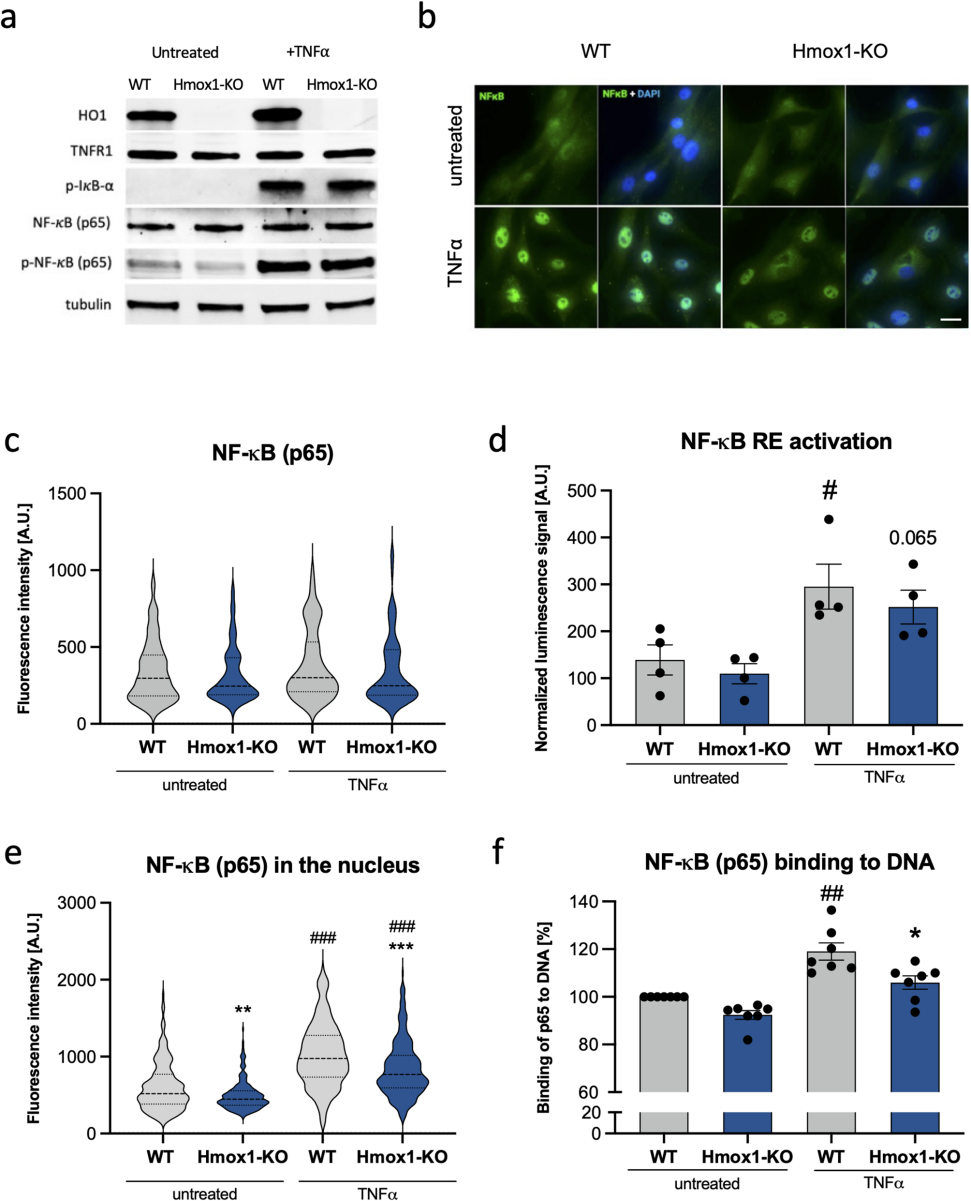
Effect of HO1 deficiency on the NF-κB signaling pathway. **a** Detection of HO1, TNFR1, phospho-IκB-α (Ser32/36), NF-κB (p65), and phospho-NF-κB (p65 – Ser536) in WT and Hmox1 KO fibroblasts either untreated or stimulated with TNFα (10 ng/mL 30 min; representative western-blot). Tubulin was used as a loading control. **b** Representative immunofluorescence images of p65 (green) and DAPI-stained nuclei (blue). Scale Bar: 20 μm. **c** Quantification of p65 protein levels in fibroblasts, untreated or stimulated with TNFα (10 ng/mL, 30 min; immunocytochemistry; *N* = 3; two-way ANOVA). **d** Transcriptional activity of NF-κB (p65) measured using a luciferase reporter system. WT and Hmox1 KO fibroblasts were treated with TNFα (100 ng/mL, 5 h; *N* = 3; two-way ANOVA). **e** Nuclear p65 levels in WT and Hmox1 KO fibroblasts after TNFα treatment (10 ng/mL, 30 min; immunocytochemistry; *N* = 3; two-way ANOVA). **f** DNA binding activity of p65 in nuclear extracts of WT and Hmox1 KO fibroblasts, untreated or treated with TNFα (10 ng/mL, 30 min; transAM ELISA; *N* = 3; two-way ANOVA). * - Hmox1 KO vs. WT, # - treated vs. untreated. Data were normalized to WT untreated cells

To better understand which aspect of NF-κB function is influenced, we compared the effects of TNFα stimulation in WT and Hmox1 KO cells. Western blot analysis (Fig. 4A) showed no differences in the total protein levels of TNF receptor-1 (TNFR1) and the NF-κB p65 subunit between WT and Hmox1 KO fibroblasts. Moreover, phosphorylation of the NF-κB inhibitor-α (IκB-α; Ser32/36) and the p65 subunit (Ser536) – a marker of NF-κB activation – was similarly robust in both genotypes 30 min after TNFα treatment (Fig. 4A). Immunocytochemical staining (Fig. 4B, C) also did not reveal any significant differences in total p65 levels, confirming the western blot results (Fig. 4A). Finally, using a luciferase reporter assay, we observed only subtle differences in NF-κB functional activity; the response to TNFα in Hmox1 KO cells, although detectable, did not reach statistical significance (Fig. 4D).

However, analysis of nuclear p65 staining revealed a reduced signal in *Hmox1*-deficient cells, both under control conditions and following TNFα stimulation (Fig. 4E). We also assessed p65 binding to specific DNA sequences in nuclear extracts from WT and Hmox1 KO fibroblasts. TransAM ELISA demonstrated that after 30 min of TNFα treatment, p65 binding to its consensus sequences was weaker in Hmox1 KO cells (Fig. 4F). We propose that this effect may reflect the reduced levels of nuclear p65 in the absence of HO1. Taken together, our results suggest impaired nuclear retention of p65 in *Hmox1*-deficient cells.

**Fig. 5:**
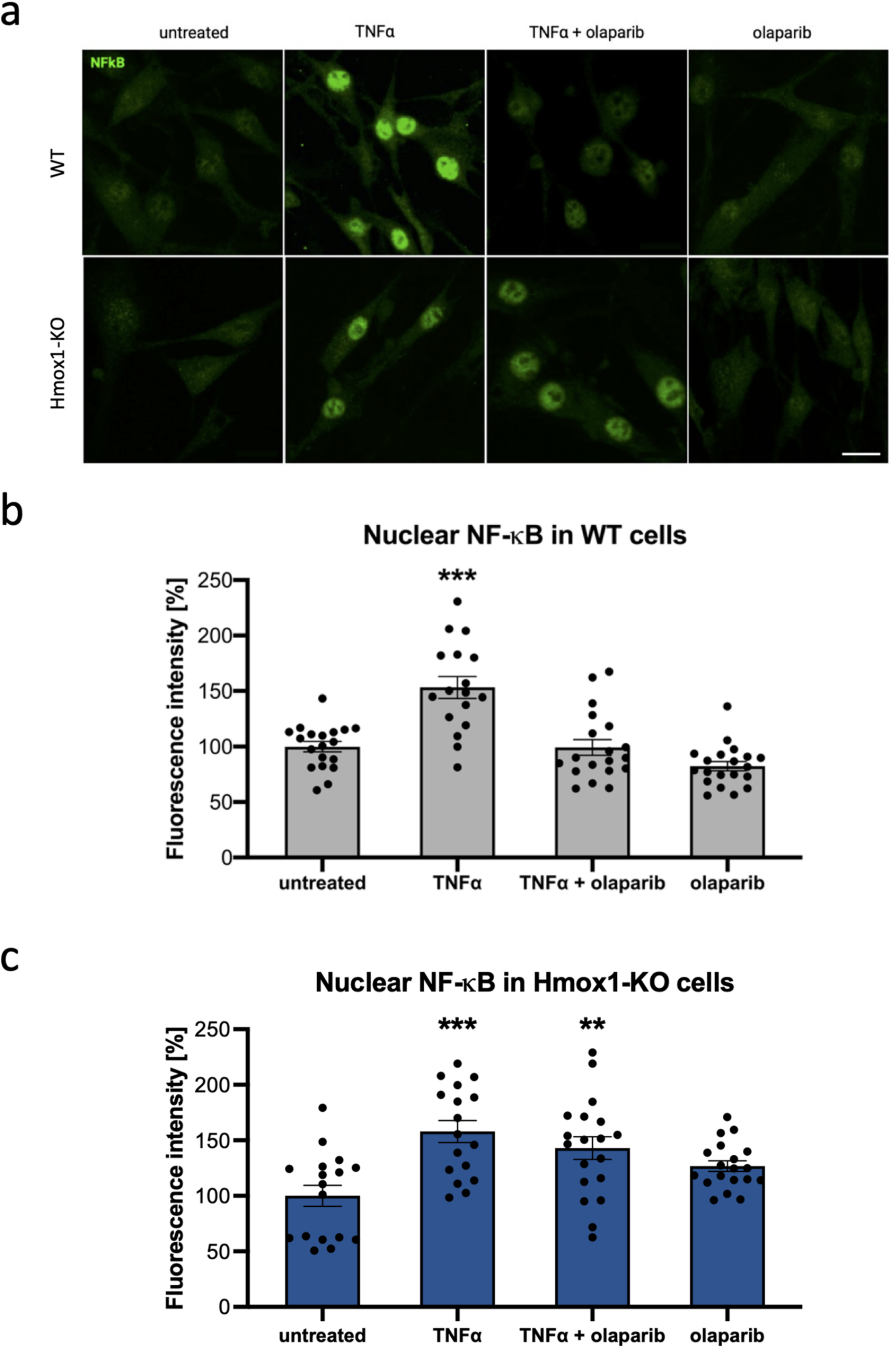
Effect of olaparib on nuclear p65.** a** Representative images of immunofluorescence staining of p65 in WT and Hmox1 KO fibroblasts (scale Bar: 20 μm). **b** Effect of olaparib on nuclear p65 levels in WT cells. **c** Effect of olaparib on nuclear p65 levels in Hmox1 KO cells. Fibroblasts were stimulated with TNFα (10 ng/mL, 30 min) or olaparib (100 nM, 90 min). *N* = 3, *n* = 17–20. ANOVA with Holm-Sidak’s test. * - treated vs. untreated. Data were normalized to WT untreated cells

### Nuclear localization of p65 is maintained by PARylation in WT but not in Hmox1 KO cells

HO1 has been reported to co-precipitate with PARP1 and to modulate both PARP1-mediated PARylation and PARG-mediated dePARylation [39, 40]. In response to proinflammatory signals, PARylation of p65 reduces its interaction with exportin CRM1, thereby facilitating nuclear accumulation of NF-κB [41]. We therefore tested whether PARylation contributes to the nuclear retention of p65 in WT and Hmox1 KO fibroblasts.

To do so, we pretreated cells of both genotypes with olaparib (a PARP1 and PARP2 inhibitor) for 1 h and then stimulated them with TNFα for 30 min (Fig. 5). In WT fibroblasts, TNFα-induced nuclear accumulation of p65 was completely abolished by olaparib pretreatment (Fig. 5A, B).

Surprisingly, in Hmox1 KO cells, inhibition of PARylation only slightly reduced the nuclear levels of p65 (Fig. 5A, C). This suggests that PARylation plays a less prominent role in regulating p65 nuclear trafficking in Hmox1 KO cells. One possible explanation is that the absence of HO1 already impairs PARP1 function, such that further inhibition has little additional effect on the already reduced nuclear localization of p65 in Hmox1 KO fibroblasts.

**Fig. 6:**
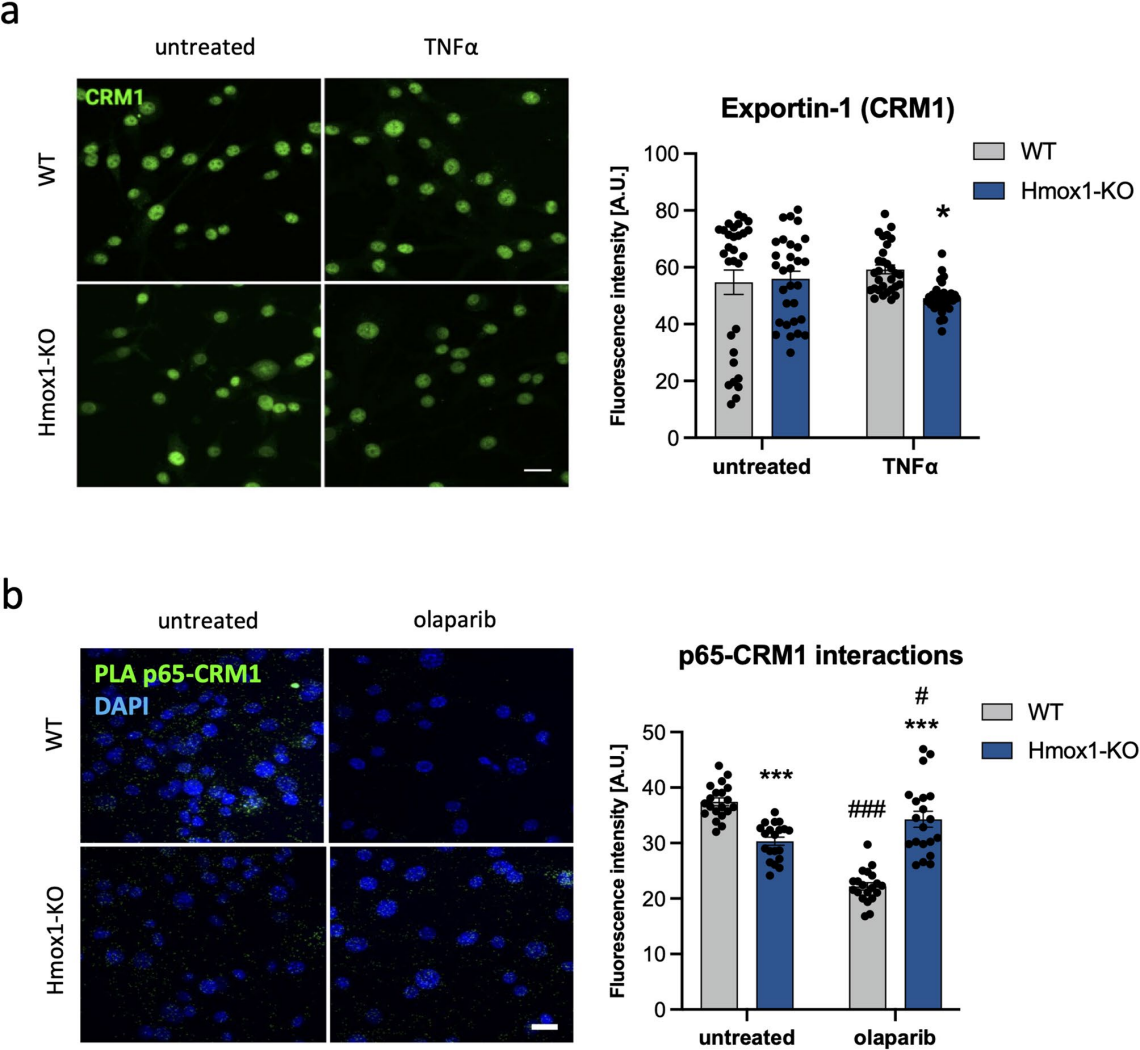
Effect of PARylation on CRM1 and p65 interaction in the cells lacking HO-1.** a** Representative immunofluorescence images of CRM1 (scale Bar: 10 μm) and quantification of CRM1 levels in fibroblasts treated with TNFα (10 ng/mL, 24 h; *N* = 3, two-way ANOVA). **b** Representative images of p65 and CRM1 colocalization (green *foci*) visualized by proximity ligation assay (PLA) in WT and Hmox1 KO fibroblasts, untreated or treated with olaparib (100 nM; 24 h). Cell nuclei were counterstained with DAPI (blue). Scale Bar: 10 μm. Right panel shows quantitative analysis of nuclear PLA signal (*N* = 3, *n* = 20; two-way ANOVA). * - Hmox1 KO vs. WT, # - treated vs. untreated

We also compared the effect of PARylation on the interaction between CRM1 and p65 in the presence and absence of HO1. Immunocytochemical staining revealed comparable nuclear levels of CRM1 in WT and Hmox1 KO cells, although after 24 h of TNFα stimulation a slight decrease was observed in *Hmox1*-deficient fibroblasts (Fig. 6A). To assess whether olaparib affects CRM1-p65 colocalization, we used a proximity ligation assay (PLA). To avoid the confounding effect of potential changes in CRM1 expression, PLA was performed in unstimulated cells. In these cells, nuclear localization of p65 was lower in the absence of HO1 (Fig. 4E), despite similar total p65 level (Fig. 4C). Accordingly, the nuclear PLA signal was also weaker in Hmox1 KO cells (Fig. 6B). Interestingly, in WT fibroblasts treated with olaparib, we observed a significant reduction in nuclear colocalization of CRM and p65, whereas *Hmox1*-deficient cells were insensitive to this treatment (Fig. 6B). These results support the conclusion that PARylation regulation of CRM1–p65 interaction is disrupted in the absence of HO-1.

### Reduced nuclear accumulation of STAT1 in Hmox1 KO cells

To determine whether the observed impairment is specific to the NF-κB pathway or reflects a broader regulatory defect, we examined the effect of *Hmox1* deficiency on the transcription factors STAT1 and STAT2, which are key mediators of the interferon response. IRF9, which was upregulated in both WT and Hmox1 KO cells in response to TNFα (Fig. 3C), forms a heterotrimeric ISGF3 complex with STAT1 and STAT2. This complex binds ISRE sequences in ISGs and mediates the late phase of the IFN-I response. In addition, STAT1 can form homodimers downstream of INFAR activation [18]. Notably, STAT1 transcriptional activity has been shown to depend on PARylation [25].

**Fig. 7:**
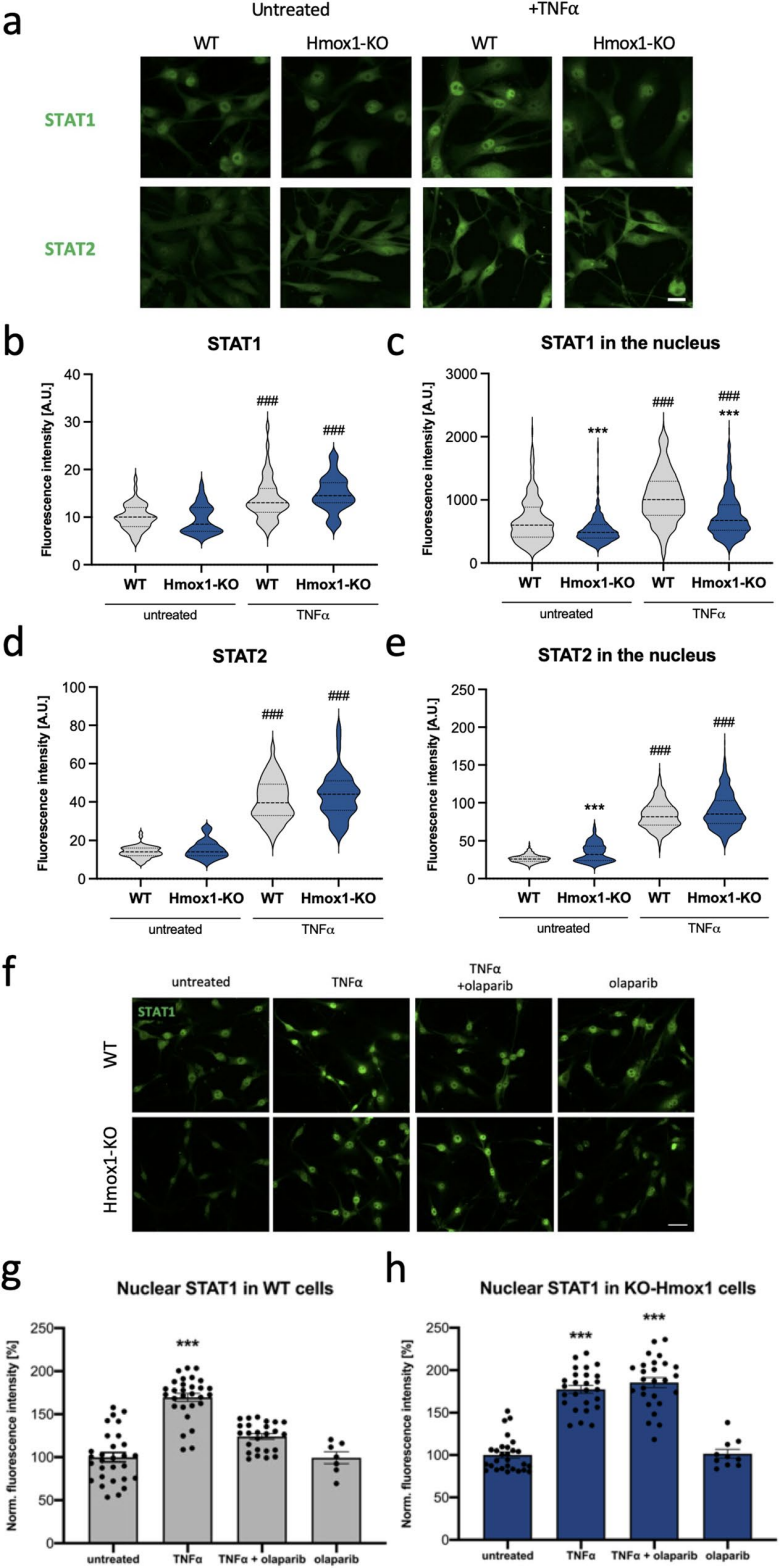
Effect of HO1 on nuclear accumulation of STAT1 and STAT2. **a** Representative images of STAT1 and STAT2 immunofluorescence staining in WT and Hmox1 KO fibroblasts under control conditions and after TNFα stimulation. Scale Bar: 20 μm. **b** Quantification of STAT1 levels in whole cells and **c** in nuclei only (*N* = 3, two-way ANOVA). **d** Quantification analysis of STAT2 levels in cells and **(e)** in nuclei only (*N* = 3, two-way ANOVA. **(f)** Representative images of STAT1 immunofluorescence staining. Scale Bar: 40 μm. **g** Quantification of nuclear STAT1 levels in WT fibroblasts and **h** Hmox1 KO fibroblasts treated with TNFα (10 ng/mL, 30 min) and olaparib (100 nM, 90 min) (*N* = 3, *n* = 28–30; ANOVA with Holm-Sidak’s test). Data were normalized to untreated cells

Total levels of STAT1 and STAT2 were similar in both genotypes under control conditions. They were also similarly increased after TNFα stimulation (Fig. 7A-C). As with p65, we observed reduced nuclear accumulation of STAT1 in both untreated and TNFα-stimulated Hmox1 KO cells (Fig. 7C). In contrast, STAT2 nuclear levels were not diminished in Hmox1 KO cells; in fact, under control conditions, nuclear staining for STAT2 appeared even stronger (Fig. 7E). Therefore, in subsequent analyses, we focused on STAT1, which displayed regulatory pattern resembling that of p65.

To investigate the role of PARylation, we preincubated cells of both genotypes with olaparib for 1 h, then stimulated them with TNFα, similarly to the p65 analyses. The results obtained (Fig. 7F–H) closely resembled those observed for p65 (Fig. 5). Specifically, TNFα-induced nuclear accumulation of STAT1 was completely blocked by olaparib pretreatment in WT cells (Fig. 7G), whereas olaparib had no effect on nuclear STAT1 in Hmox1 KO cells (Fig. 7H). Thus, the nuclear accumulation of some transcription factors, such as NF-κB and STAT1, relies on different mechanisms in *Hmox1*-competent and *Hmox1*-deficient cells. In the absence of HO1, cells depend less on PARylation-regulated processes.

### Hmox1-deficiency increases envelope permeability

**Fig. 8:**
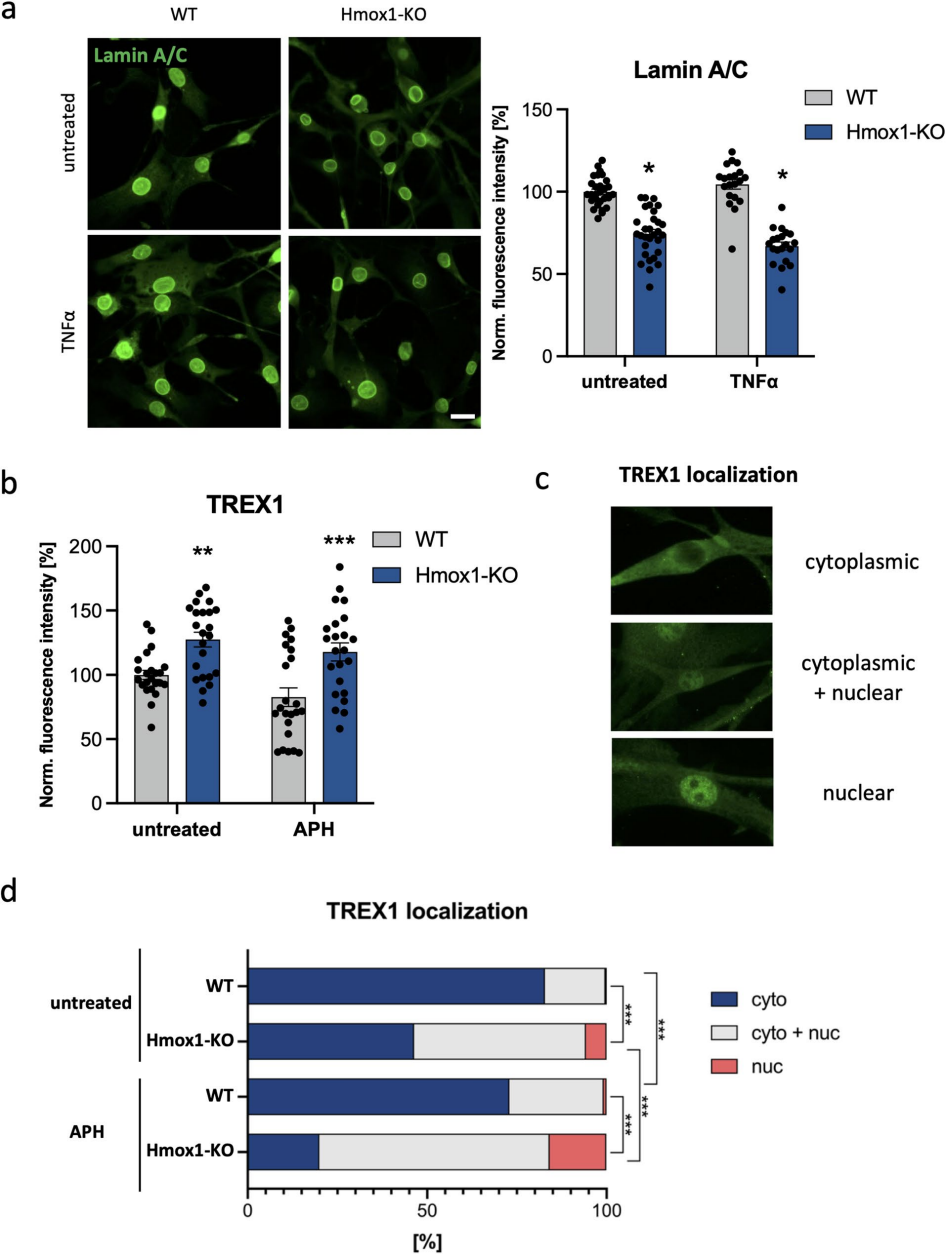
Effect of HO1 on lamin A/C and TREX1. **a** Representative images and quantitative analysis of immunofluorescence staining of lamin A/C in fibroblasts untreated and treated with TNFα (10 ng/ml, 24 h: *N* = 3, two-way ANOVA. Data were normalized to WT untreated cells. **b** Quantification of immunofluorescence staining of TREX1 in fibroblasts untreated or treated with APH (10 ng/mL, 24 h; *N* = 3, two-way ANOVA). Data were normalized to WT untreated cells. **c** Representative images of TREX1 depicting protein localized in: cytoplasm only (cyto), both in cytoplasm and nucleus (cyto + nuc) and only in the nucleus (nuc). **d** The percentage of cells expressing TREX1 protein in specific cell compartments of WT and Hmox1 KO fibroblasts untreated or treated with APH (10 ng/mL, 24 h) (*N* = 3, Chi^2^ test)

Our results indicate that *Hmox1* deficiency affects the nuclear accumulation of certain transcription factors, possibly through multiple mechanisms, including dysregulation of PARylation-dependent pathways. Macromolecular transport between the nucleus and cytoplasm is regulated not only by the nuclear pore complex, but also by structural components of the nuclear envelope, including the nuclear lamina [42, 43]. Both direct and indirect crosstalk exists between PARylation and nuclear envelope proteins, with lamin A/C being one of the known targets [44, 45]. Therefore, we examined whether *Hmox1* deficiency could also affect lamin A/C levels. Indeed, immunofluorescence staining revealed reduced level of lamin A/C in Hmox1 KO cells, both under control conditions and after TNFα treatment (Fig. 8A). In light of this and the observed alterations in CRM1 function (Fig. 6B), we hypothesized that Hmox1 deficiency might generally increase nuclear envelope permeability.

The nuclear localization of TREX1 exonuclease has emerged as an indicator of nuclear envelope permeability, particularly in the context of cellular stress and DNA damage. Under physiological conditions, TREX1 is associated with the endoplasmic reticulum, and its presence in the nucleus may reflect compromised nuclear envelope integrity [46]. We have recently demonstrated that a universal consequence of *Hmox1* deficiency is replication stress, observed, among others, in HSCs isolated from the bone marrow of *Hmox1* KO mice, as well as in lymphoblastoid cells from an *HMOX1*-deficient patient [3]. In addition, we observed an increased DNA damage response (DDR), indicated by γH2AX staining, in Hmox1 KO fibroblasts, both under control conditions and after induction of genotoxic or replication stress using etoposide or aphidicolin, respectively (Fig. S2A).

We performed immunocytochemical staining for TREX1 in WT and Hmox1 KO cells (Fig. 8B). The total TREX1 level was elevated in unstimulated *Hmox1*-deficient cells compared to WT counterparts. A similar increase was observed in cells subjected to replication stress, induced by aphidicolin (Fig. 8B). Notably, TREX1 is an interferon-stimulated gene (ISG) and its expression is upregulated in response to cytosolic DNA [47]. In contrast, CGAS and STING1, two other components of this pathway, were unaffected (Fig. S2B, C).

Next, we analyzed TREX1 localization. We classified its distribution into three categories (Fig. 8C): (i) predominantly cytoplasmic (cyto); (ii) both cytoplasmic and nuclear (cyto + nuc); (iii) predominantly nuclear (nuc). As expected, in WT fibroblasts TREX1 was primarily localized to the cytoplasm (80% of cells). In response to aphidicolin, the proportion of cyto + nuc cells significantly increased (Fig. 8D), although the percentage of cells with predominantly nuclear TREX1 (nuc) remained negligible. Interestingly, in Hmox1 KO cells under control conditions, TREX1 was exclusively cytoplasmic (cyto) only in 46% of cells, whereas in 48% it was localized both in the cytoplasm and nucleus, and in 6% predominantly in the nucleus (Fig. 8D). Aphidicolin treatment further increased the cyto-nuc fraction to 64% and the nuclear only fraction to 16%. These observations support the notion that HO1 deficiency is associated with increased permeability of the nuclear envelope.

## Discussion

In previous studies, we observed that in infection-free *Hmox1* KO mice, expression of interferon-stimulated genes (ISGs) is elevated in various cell types, despite unchanged interferon levels [8, 9, 48]. Current findings lead to the conclusion that while cell-autonomous upregulation is possible for some genes (e.g., *Ifi27*), their expression in most cases is regulated extrinsically in vivo – most likely as a response to pre-inflammatory state and increased production of proinflammatory cytokines, characteristic of Hmox1 KO mice.

Paradoxically, under inflammatory conditions induced by exogenous TNFα in vitro, we found that the response of *Hmox1*-deficient cells is attenuated. Our results show that HO1 expression promotes nuclear envelope integrity, facilitates nuclear retention of NF-κB and STAT1, and preserves sensitivity to PARylation-dependent regulation of nuclear-cytoplasmic transport.

HO1 is a heme-degrading enzyme induced in response to oxidative stress and inflammation. For years, it has been described as cytoprotective and anti-inflammatory – both due to the biological activity of its catabolic products and through direct interaction with proinflammatory transcription factors [38]. In mice, *Hmox1* deficiency leads to microcytic, hemolytic anemia, already observed in pups and exacerbated with age, as evidenced by a very low level of circulating hepcidin [49]. This phenotype is primarily caused by disturbed iron metabolism and the depletion of CD163^+^ macrophages, which are responsible for the clearance of senescent erythrocytes and are typically reduced in Hmox1 KO mice [15, 16]. Importantly, during hemolytic stress, heme released into the extracellular space can bind as a partial agonist to membrane TLR4, thereby activating both NF-κB-dependent inflammation and the IRF-dependent IFN-I response [50].

TLR signaling can be classified as either MyD88-dependent, which drives the induction of inflammatory cytokines, or TRIF-dependent, which is responsible for the induction of the IRF-I response as well as inflammatory cytokines. TLR4 is the only TLR that activates both the MyD88- and TRIF-dependent pathways [36]. It activates IRF3, IRF7, or IRF8, thereby inducing ISG expression [51]. We initially suspected that the increased ISG expression observed in Hmox1 KO mice might result from hemolytic stress and TLR4 activation by heme.

In this study, to examine the effect of hemolytic stress on ISG expression, we stimulated WT and Hmox1 KO fibroblasts cultured in vitro with hemoglobin. Additionally, we used 5-aminolevulinic acid (ALA), a heme synthesis precursor, to assess the influence of intracellular free heme overload. We have recently shown that the absence of HO1 increases the pool of labile heme following ALA treatment [3]. Interestingly, we did not observe any changes in ISG expression in response to either Hb or ALA, suggesting that hemolytic stress or increased labile heme are probably not responsible for the upregulation of ISGs in vivo. Furthermore, neither the induction of the DNA damage response nor that of replication stress – both of which are enhanced in HO1-deficient HEK293 cells [3] and murine bone marrow-derived HSCs [5] – induced ISG expression in mouse primary fibroblasts, regardless of HO1 status.

HO1 reduces oxidative stress by removing excess free heme, which can be prooxidant. However, untreated WT and Hmox1 KO fibroblasts showed similar levels of ROS and lipid peroxidation, and additional oxidative stress did not induce ISG expression. This resembles the pattern we observed in WT and HMOX1 KO HEK293 cells [3]. Moreover, bone marrow mesenchymal stromal cells isolated from Hmox1 KO mice were able to rapidly activate antioxidant defenses when challenged with hemin [52]. This suggests that oxidative stress is not the main driver of the IFN-I response. The only stressor that induced ISG expression (*Irf1*,* Irf7*,* Irf9*,* Ifitm3*,* Oas1g*,* Oasl1*) in fibroblasts cultured in vitro was TNFα, a proinflammatory cytokine. Therefore, we believe that ISG induction in Hmox1 KO mice results from a pre-inflammatory state and increased production of pro-inflammatory cytokines, including TNFα.

The involvement of HO1 in the regulation of interferon response has been reported previously. Specifically, HO1 was shown to be required for TLR4-induced production of IFN-I and expression of IRF3 target genes in macrophages [19]. IRF3 exists in two forms, a monomeric form that is mainly exported to the cytoplasm, and a phosphorylated dimeric form that is retained in the nucleus. Interestingly, HO1 was suggested to directly interact with IRF3, promoting its phosphorylation and subsequent nuclear retention [19]. Although the underlying mechanism is different, the increased nuclear accumulation of IRF3 in the nucleus is consistent with our findings showing enhanced nuclear retention of NF-κB, STAT1 (present study), and p53 [3], suggesting a more general role for HO1 in regulating nucleo-cytoplasmic trafficking.

The modulatory effect of HO1 may result from distinct, not mutually exclusive mechanisms. HO1 was recently shown to enhance IFN-I signaling during viral infections, possibly through increased nuclear retention of IRF3 [20]. In contrast, it inhibited IFN-I and ISRE promoter activation following H_2_O_2_ treatment, due to the promotion of IRF3 degradation via an autophagosome-dependent pathway. This effect was dependent on HO1 enzymatic activity and iron availability [20]. However, in our experimental setting, we did not observe any effect of H_2_O_2_ on ISG expression.

It is well established that stimulation of human fibroblasts or macrophages with TNFα significantly enhances the IFN-I response [53]. However, the reciprocal regulation between the IFN-I pathway and inflammation is cell-type dependent [54]. Notably, Hmox1 KO mice exhibit elevated levels of circulating MCP1, TNFα, and several other proinflammatory cytokines, whereas increased HO1 expression – resulting from a polymorphism in the *HMOX1* promoter – confers greater resistance to oxidative stress and attenuates the inflammatory response in humans [55]. This suggests that the net effect of HO1 is to mitigate inflammation. However, the regulation of individual components of the inflammatory response may be more complex and highly cell-type specific.

Furthermore, iron – the end-product of HO-1 enzymatic activity – has been shown to inhibit NF-κB nuclear translocation in primary prostate cells [56]. Iron was also reported to suppress p65 phosphorylation, thereby reducing NF-κB activity in endothelial cells [57]. Docking analysis suggested that HO-1 may directly interact with p65 and decrease its DNA-binding capacity [38]. Notably, however, no significant effect of HO1 on NF-κB was observed in macrophages [19].

Interestingly, primary mouse Hmox1 KO fibroblasts cultured in vitro showed a weaker response to exogenous TNFα stimulation. This observation initially suggested a disturbance in TNF receptor signal transduction in the absence of HO1. However, western blot and immunocytochemical staining revealed no significant differences in TNFR levels or in total and phosphorylated p65. The key difference between WT and Hmox1 KO cells was the subcellular localization of the p65 subunit of NF-κB, which normally translocates to the nucleus and activates the expression of INF-I genes in response to TNFα. Analysis of p65 localization and DNA binding indicated impaired nuclear accumulation and function of p65 in the nuclei of Hmox1 KO cells. This could potentially result from abnormal nuclear transport – including both import and export – or from a faulty nuclear envelope.

A key universal regulator of transport between the nucleus and the cytoplasm is PARylation. It has been reported that PARP1 and NF-κB form a stable, immunoprecipitable nuclear complex [58, 59] and that PARylation of p65 is a critical determinant for its interaction with the nuclear export protein CRM1, thereby promoting nuclear retention of NF-κB [60]. However, this mechanism may not be universal: in *Trypanosoma cruzi*-infected cardiomyocytes, direct interaction between PARP1 and p65 was not observed. Instead, PARP1-mediated PARylation of p65-interacting nuclear proteins enhanced NF-κB activation and cytokine gene expressions [59]. Notably, inhibition of PARylation reduced nuclear localization of p65 in such cells [59], as well as in smooth muscle cells stimulated via TLR4 [61] – an effect closely resembling what we observed in wild-type fibroblasts treated with olaparib.

Moreover, the pattern we detected for STAT1 closely mirrored that of p65. Specifically, HO1 enhanced nuclear localization of STAT1 in primary mouse fibroblasts, which is consistent with a previous report showing increased STAT1 activity in *Hmox1*-expressing cells [6]. As with p65, inhibition of PARylation abolished the TNFα-induced nuclear retention of STAT1. These findings suggest that the regulatory role of PARylation is not limited to a single transcription factor, but rather represents a more general mechanism.

Indeed, PARP-1 has been implicated in controlling the subcellular localization of NF-κB, STAT1, and p53 [61, 62]. No direct relationship has been identified between PARP1 and components of the importin system, such as importin a3 and a4 [61]. In contrast, PARP1 expression and activity influence cytosolic levels of CRM1 and the dynamics of its trafficking, which is required for the nuclear export of NF-κB, p53, and STAT1 [61]. PARylation of p53 or p65 NF-κB by PARP1 reduces their interaction with CRM1, thereby rendering these transcription factors resistant to export and promoting their retention in the nucleus [41, 61, 62]. We observed that olaparib markedly affects the colocalization of p65 and CRM1 in wild-type fibroblasts. However, the functional consequences of these changes, as measured 24 h after olaparib treatment, remain difficult to interpret unambiguously. Unexpectedly, the effects of olaparib on TNFα-induced nuclear retention of p65 and STAT1, as well as p65-CRM1 colocalization, were completely abolished in the absence of HO1.

Thus, a model has emerged in which PARylation of stress-responsive transcription factors prevents their nuclear export, thereby promoting their accumulation in the nucleus [41]. Our results suggest that these regulatory pathways are impaired in *Hmox1*-deficient cells. At this point, we can only speculate about the underlying cause of this phenomenon. Interestingly, HO1 has been shown to co-precipitate with PARP1, indicating a potential direct protein-protein interaction [40]. Based on docking models and pull-down experiments, HO1 is proposed to bind to the regulatory helical domain (HD) of PARP1 [40], potentially inducing an open conformation that leaves PARP1 in a constitutively active state [40]. Additionally, HO1 may interact with PARG protein, inhibiting its dePARylating activity [63]. These observations suggest that, in the absence of HO1, the PARylation level may be lower, which could consequently facilitate the export of transcription factors from the nucleus.

However, our recent studies did not detect such a relationship. Proximity ligation assay confirmed a colocalization of PARP1 and HO1, yet PARP1 accumulated at the sites of DNA damage more rapidly in the absence of HO1 [3]. Similarly, PAR formation at sites of laser micro-irradiation occurred earlier in HO1-deficient cells. On the other hand, autoPARylation of purified PARP1 protein was comparable in the presence or absence of HO1. These findings suggest that the effects of HO1 deficiency on PARylation are more likely related to altered PARP1 cellular motility, than to substrate availability or a direct protein-protein interaction modulating PARP1 enzymatic activity [3]. Additionally, we demonstrated that the effects of HO1 on p53 are more strongly associated with the regulation of free heme than with PARylation [3].

Another potential trigger of IFN-I response and ISG expression is the presence of cytosolic DNA, generated intrinsically during cell proliferation and DNA repair. This DNA typically Ranges in length from 100 to 1000 nucleotides, and the IFN-I pathway can be activated by fragments as short as 24 nucleotides, regardless of sequence [61, 62]. Replication stress and fork stalling, which activate the DNA damage response (DDR), appear to be the main intrinsic sources of cytoplasmic DNA. Recently, we demonstrated that HO1 co-localizes with DNA G4 structures both in the nucleus and cytoplasm and plays a role in regulation of G4 unwinding [5]. Moreover, loss of *Hmox1* in proliferating cells is associated with enhanced G4 formation and replication fork stalling [3]. In the present study, Hmox1 KO fibroblasts exhibited increased DDR under basal conditions and after stimulation with etoposide (genotoxic stress) or aphidicolin (replication stress). Unfortunately, we were unable to reliably compare cytoplasmic DNA levels between WT and Hmox1 KO fibroblasts. However, experiments in murine iPS cells revealed that HO1 deficiency leads to elevated levels of cytoplasmic DNA following aphidicolin stimulation [data not shown].

Finally, abnormal nuclear localization of key transcription factors as well as cytoplasmic leakage of DNA may result from deregulated nuclear envelope permeability. The inner membrane of nuclear envelope is stabilized by lamins, while the outer membrane forms a continuum with the endoplasmic reticulum [64], which is the primary cellular localization of HO1. In laminopathies, pore disintegration during interphase leads to disturbed protein localization [65]. Our data show that *Hmox1*-deficient cells have lower levels of lamin A, a crucial protein for maintaining nuclear structure and mechanical stability.

A marker of impaired nuclear envelope integrity may be the presence of the TREX1 exonuclease in the nucleus [46], where it cleaves chromatin, leading to DNA damage [66]. In *Hmox1*-deficient cells, TREX1 levels are increased, which may contribute to elevated dsDNA breaks. Importantly, Hmox1 KO fibroblasts also showed higher nuclear localization of TREX1, suggesting disturbances in nuclear envelope integrity and potentially additional DNA cleavage.

## Summary

In conclusion, our results indicate that the increased ISG expression previously reported in *Hmox1*-deficient mice is driven primarily by extrinsic, organism-level factors, likely by elevated production of proinflammatory cytokines such as TNFα. At the cellular level, *Hmox1* deficiency results in a diminished response to TNFα and impaired nuclear retention of NF-kB and STAT1. It is also associated with a decreased reliance on PARylation for regulating transcription factor trafficking, and with increased susceptibility to nuclear envelope disruption.

## Supplementary Information

Supplementary Material 1.

## Data Availability

Data will be made available on request.
