# Supplementary material for: Effect of heme oxygenase-1 on the expression of interferon-stimulated genes
Source: J Inflamm (Lond). 2025 Oct 9;22:43. doi: 10.1186/s12950-025-00467-5 (PMC12512898; doi:10.1186/s12950-025-00467-5)
Supplement: Supplementary file 1 — Supplementary Material 1. [file 12950_2025_467_MOESM1_ESM.pdf]

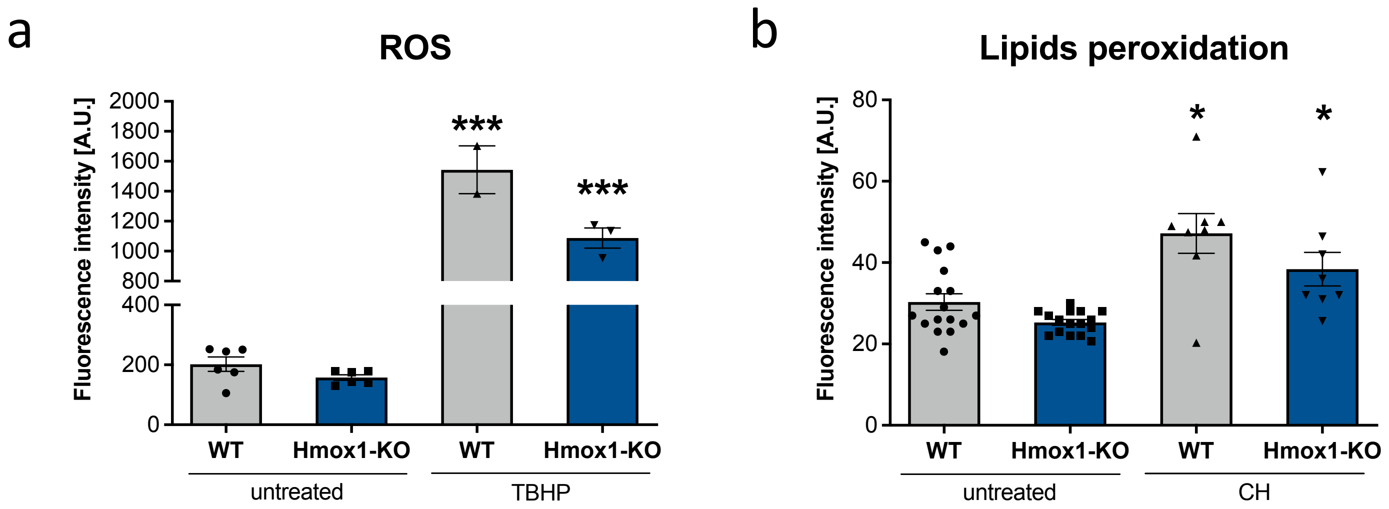

Fig. S1 a) ROS levels in WT and KO-Hmox1 fibroblasts. TBHP (200 µM, 30 minutes) was used as a positive control. N=3. Two-way ANOVA. **b)** Lipids peroxidation in WT and KO-Hmox1 fibroblasts. Cumene hydroperoxide (CH, 100 µM, 24 h) was used as a positive control. N=3. Two-way ANOVA.

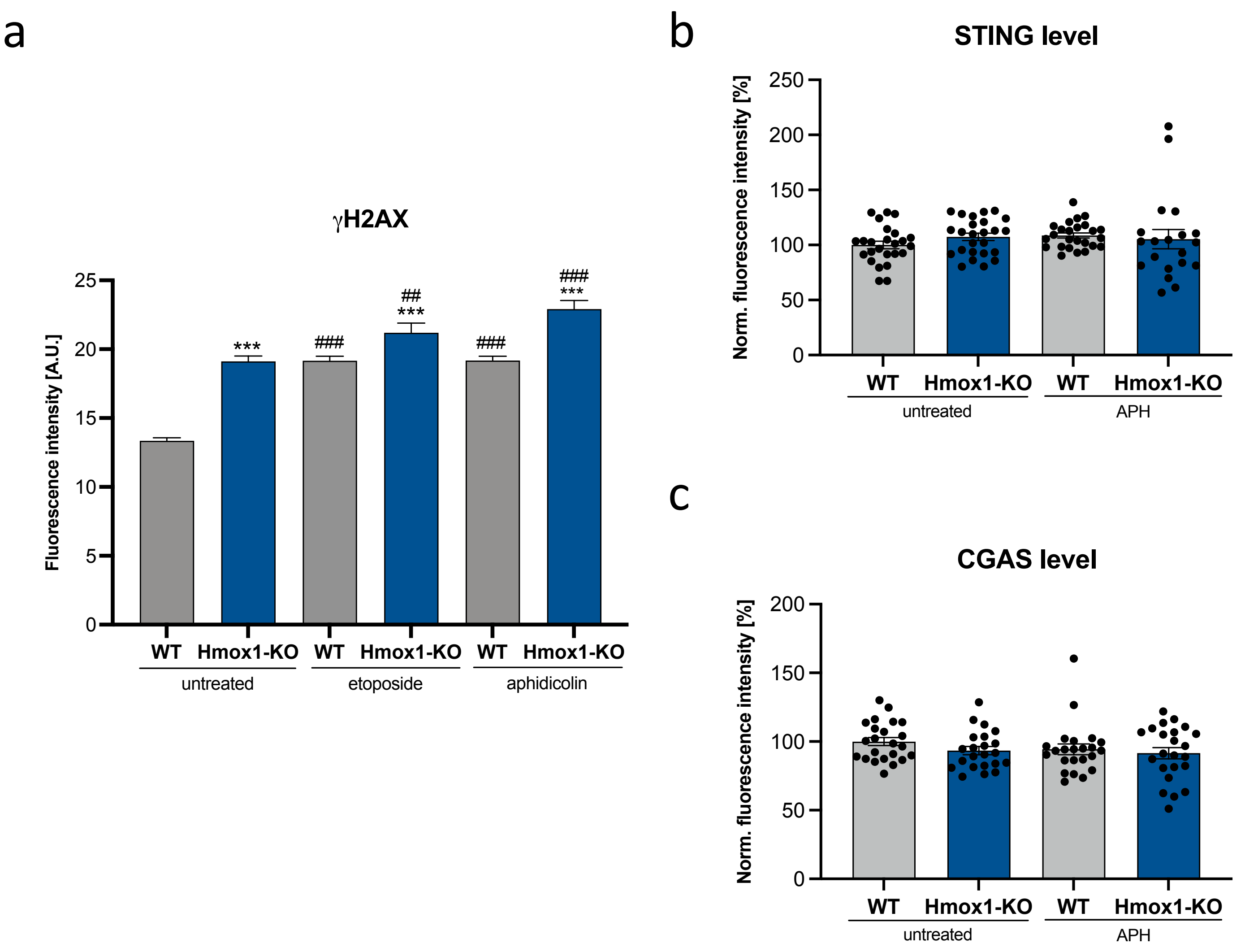

Fig. S2 a) Quantitative analysis of ɣH2AX fluorescence signal in fibroblasts treated with 0.25 µM etoposide or 0.01 µg/ml aphidicolin for 24h. N=3, n=326-792. Two-way ANOVA. * - WT vs KO-Hmox1, # - untreated vs treated. **b)** Quantitative analysis of of immunofluorescence staining of STING and **(C)** CGAS in fibroblasts treated with 0.01 µg/ml aphidicolin (APH) for 24h. N=3. Two-way ANOVA.
